# The Use of Dual Reconstruction Plates for Failed Fixation of Patellar Fracture after Total Knee Replacement: A Case Report

**DOI:** 10.5704/MOJ.1611.012

**Published:** 2016-11

**Authors:** P Sa-ngasoongsong, K Chulsomlee, S Wongsak, C Suphachatwong, V Kawinwonggowit

**Affiliations:** Department of Orthopaedics, Faculty of Medicine Ramathibodi Hospital, Mahidol University Bangkok, Thailand

**Keywords:** Patella plating, periprosthetic fracture, dual plate, patellar fracture, total knee replacement

## Abstract

Patellar fracture after total knee replacement (TKR) is one of the challenging problems in periprosthetic fracture. Open reduction with internal fixation (ORIF), as tension band wiring (TBW), usually required in cases with extensor mechanism disruption. However, many studies reported a high failure rate after using this technique. In this report, we presented an interesting case of periprosthetic patellar fracture after TKR with TBW failure that was successfully treated with double non-locking reconstruction plates fixation and TBW augmentation.

## Introduction

Periprosthetic patellar fracture (PPF) is the second most common fracture following total knee replacement (TKR) with the prevalence of 0.7-1.2%. Predisposing factors for PPF include patellar osteonecrosis, limb or component mal-alignment, design of patellar component, lateral release and excessive resection of bone^1^. Previous systematic review studies demonstrated that non-operative treatment would be appropriate for the cases with minimal displacement and stable implant fixation^1, 2^. However, if there were a disruption of extensor mechanism or loosening of patellar component, surgical management was recommended. Surgical options were open reduction and internal fixation (ORIF), patelloplasty, partial patellectomy with reattach the tendon to the remaining bone and total patellectomy according to fracture type and stability of the implant^2^. However, the outcome after these methods was still unconvincing as 50% complication rate and 42% re-operation rate^2^, and therefore; ORIF with tension band wiring (TBW) was not recommended as the first choice of treatment due to a high rate of fixation failure, and risk of nonunion^1^. The aim of this case report was to demonstrate a case of PPF after TKR and complicated with TBW failure that was successfully treated with ORIF using double reconstruction plates fixation and TBW augmentation. 

## Case Report

**Fig. 1:**
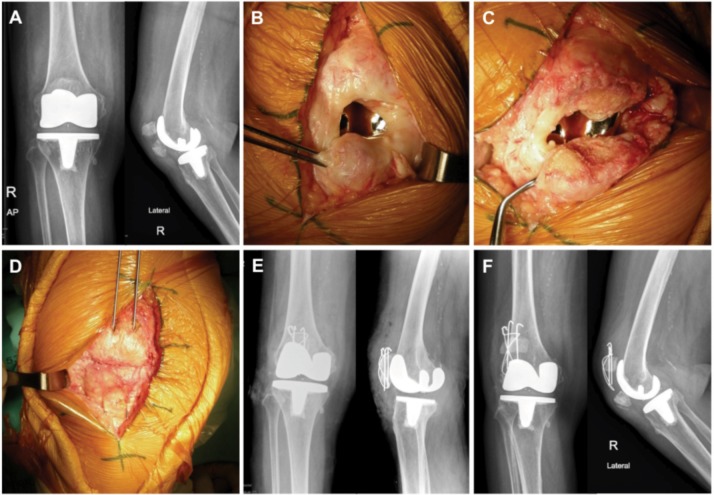
Initial radiographs, intraoperative findings, and postoperative radiographs after the first operation; (A) Preoperative radiographs of right knee revealed transverse periprosthetic patellar fracture with 2-cm fracture displacement. Intraoperative findings showed displaced fracture without callus formation (B), then the fracture bed was prepared (C) and anatomically fixed with tension band wiring (D). Postoperative radiographs on the first day (E) after open reduction and internal fixation with tension band wiring, and one-month postoperative radiographs (F) showed displaced fracture with failed implant fixation.

A 67 years old female presented with closed transverse right patellar fracture after simple fall on her knee two months previously (Fig. 1A) and gave a history of patellar nonresurfacing TKR 9 years ago. Examination revealed tenderness, 2-cm fracture gap with 45-degree extension lag, and inability to actively extend the right knee. Open reduction and internal fixation with TBW and repair of the ruptured extensor retinaculum was performed. The intraoperative findings were a simple transverse patellar fracture without callus formation, and well-fixed TKR implants (Fig. 1B). The fibrous tissue was removed and the fracture was reduced anatomically under compression with TBW (Fig. 1C-E). Routine postoperative care and rehabilitation protocol were then applied. However, on the first follow-up month, the patient reported persistent pain and inability to fully extend the knee, and examination revealed a 1.5-cm palpable fracture gap with 30-degree extension lag. The 1-month postoperative radiographs demonstrated displaced patellar fracture with cutting-out wire loop and superior K-wires migration (Fig. 1F). Laboratory investigation showed normal erythrocyte sedimentation rate and C-reactive protein level. The diagnosis was peri-prosthetic patellar fracture with early fixation failure.

Cause analysis and preoperative planning for revision surgery were discussed. We hypothesized that the causes of TBW failure were multifactorial, as from poor vascular supply from previous TKR, osteoporotic bone quality, high patellofemoral force after TKR^3^, and delayed presentation, which resulted in delayed fracture healing and suboptimal fixation strength with TBW. The implant therefore could not tolerate the physiologic load from postoperative rehabilitation program and leading to early fixation failure. Considering the good bone stock from non-resurfacing patella in this case, we decided to revise the fixation with dual 3.5-mm reconstruction plates (medial and lateral sides) plus TBW. The reasons for using this plate were: (1) easy to anatomically contour along the patella, (2) allows adjustment of screw trajectory to avoid screw jamming problems, and (3) inexpensive.

**Fig. 2:**
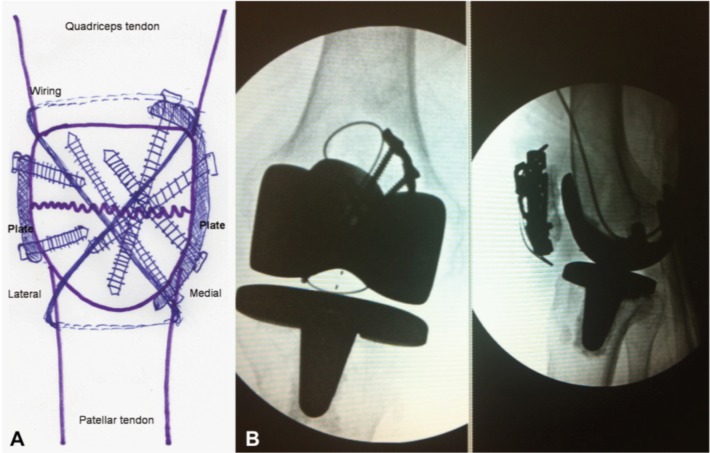
Preoperative surgical planning and intraoperative fluoroscopic images; Preoperative surgical template for open reduction with dual reconstruction plates and tension band wiring (A), and intraoperative images demonstrated successful fracture compression with stable fixation (B).

**Fig. 3:**
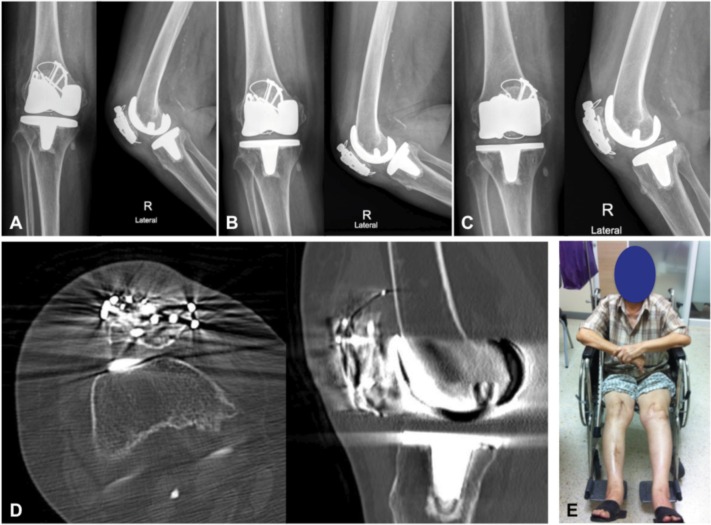
Postoperative radiographs and outcome; Postoperative follow-up radiographs on the first day (A), six months (B), and eighteen months (C) showed stable fixation without fracture displacement. On 6-month follow-up period, computerized tomography (D) demonstrated fracture healing as cortical continuation and disappearance of fracture line and the patient could fully extend her right knee similar to the normal side (E).

The surgical techniques were as follows. Under general anesthesia, the patient was placed in supine position with pneumatic tourniquet on proximal thigh. Midline incision and medial parapatellar arthrotomy approach were done. Previous TBW was removed and the fracture site was identified. The interposing fibrous tissue was removed and sent for culture. The fracture was then anatomically reduced under compression load with point reduction forceps, temporarily fixed with K-wires and checked with the fluoroscope. Then two 3.5-mm non-locking reconstruction plates (3-hole and 5-hole reconstruction plates, Synthes, Paoli, PA, USA) were contoured along the medial and lateral border of the patella, on the mid antero-posterior position (Fig. 2A). Six of 3.5-mm cortical screws were then inserted across the fracture site, under fluoroscope, in order to achieve the best possible fixation strength. The temporary K-wires were then removed and TBW augmentation was performed in full knee extension (Fig. 2B). The stability of fixation was checked under passive knee flexion (0-130 degree). Intra-articular drain tube was inserted and the extensor retinaculum was repaired followed by subcutaneous and skin closure. Compressive dressing was applied before tourniquet removal. The operation time was 80 minutes. The drain and compressive dressings were removed at 48 hours postoperatively. The tissue culture was negative. The patient was allowed to perform isometric knee exercise as soon as possible. Hinge knee brace was also applied postoperatively to restrict knee motion, to only 0-90 degree, during the first four weeks. Full weight bearing with gait aid was allowed after three months postoperatively and then followed by training on ambulation without gait aid. Deep knee flexion daily activities, such as climbing stairs and sitting on toilet, were allowed at 6-month postoperatively. Fracture healed uneventfully and the patient could walk independently after four months postoperatively. Follow-up radiographs and computerized tomography at 6-months postoperatively, proved fracture healing as shown in Fig. 3A-D. Examination on the postoperative 6-month follow-up revealed active knee extension with 0-130 degree flexion arch and grade V quadriceps muscle power without extension lag (Fig. 3E).

This case report has been approved by our Institutional Board Review, based on the Declaration of Helsinki. The patient was informed that data from the case would be submitted for publication and gave her consent.

## Discussion

Surgical management of displaced peri-prosthetic patellar fracture after total knee arthroplasty with extensor mechanism disruption could be problematic. Previous systematic review studies recommended not using ORIF with TBW as the first choice of treatment due to the high rate of fixation failure and risk of nonunion^1, 2^. In our case, we used double 3.5-mm reconstruction plates and TBW augmentation for revision surgery after TBW failure due to inadequate bone stock in previous non-resurfacing patella TKR and better fixation stability than traditional TBW as proven in previous studies^4, 5^. As a result, the fracture was stable enough to allow early postoperative range-of-motion program and able to tolerate the significant load during the fracture healing process. Therefore, we believed that this fixation technique would be preferred to the traditional TBW, and should be an alternative surgical option in this type of fracture. However, further studies are required and careful pre-operative planning, especially in the patients with poor remaining bone stock such as patellar resurfacing TKR with loosening of implant.
